# Preparation of Biocomposites with Natural Reinforcements: The Effect of Native Starch and Sugarcane Bagasse Fibers

**DOI:** 10.3390/molecules27196423

**Published:** 2022-09-29

**Authors:** Muriel Józó, Róbert Várdai, András Bartos, János Móczó, Béla Pukánszky

**Affiliations:** 1 Laboratory of Plastics and Rubber Technology, Department of Physical Chemistry and Materials Science, Budapest University of Technology and Economics, Műegyetem rkp. 3, H-1111 Budapest, Hungary; 2 Institute of Materials and Environmental Chemistry, Research Centre for Natural Sciences, P.O. Box 286, H-1519 Budapest, Hungary

**Keywords:** biopolymers, biocomposites, reinforcements, adhesion, micro-mechanics, acoustic emission

## Abstract

Biocomposites were prepared from poly(lactic acid) and two natural reinforcements, a native starch and sugarcane bagasse fibers. The strength of interfacial adhesion was estimated by model calculations, and local deformation processes were followed by acoustic emission testing. The results showed that the two additives influence properties differently. The strength of interfacial adhesion and thus the extent of reinforcement are similar because of similarities in chemical structure, the large number of OH groups in both reinforcements. Relatively strong interfacial adhesion develops between the components, which renders coupling inefficient. Dissimilar particle characteristics influence local deformation processes considerably. The smaller particle size of starch results in larger debonding stress and thus larger composite strength. The fracture of the bagasse fibers leads to larger energy consumption and to increased impact resistance. Although the environmental benefit of the prepared biocomposites is similar, the overall performance of the bagasse fiber reinforced PLA composites is better than that offered by the PLA/starch composites.

## 1. Introduction

The interest in bioplastics has increased enormously in recent years [1,2]. They are used in a wide range of applications including packaging [3,4], agriculture [5,6], and various consumer goods [7], but also as technical parts in the automotive or machine industry [8,9,10,11]. The increasing use of biopolymers is adequately justified by the huge amount of plastic waste accumulating each year, but also by other environmental concerns like microplastic pollution [12,13]. Biopolymers have several advantages including the use of renewable resources, compostability, advantageous carbon footprint, etc. On the other hand, these materials also possess some drawbacks, like somewhat inferior properties compared to commodity polymers, frequent complications during their conversion, sensitivity to water during processing [3], and higher price.

Presently, the biopolymer produced and used in the largest quantity is poly(lactic acid) (PLA). Its stiffness and strength compete even with those of engineering plastics, but its physical ageing is fast because of its low glass transition temperature [14] and its impact resistance is also rather small [15]. Accordingly, PLA is frequently modified in various ways including plasticization [16,17,18], blending [19,20,21,22,23], and the addition of reinforcements [24,25,26,27,28,29,30,31] to further improve stiffness and strength. In order to maintain the inherent advantages of biopolymers of small carbon footprint, natural origin, and compostability, they are often combined with bio-based additives [32,33,34,35,36,37,38,39,40] such as reinforcements from natural resources. Wood flour [24,25,28] and various natural fibers [26,30,31] are used the most frequently, but microcrystalline [29] or regenerated cellulose and lignin [19] are also added to PLA to modify its properties.

Starch is also a biopolymer produced by plants for energy storage and it is available in large quantities [41]. Because of its large molecular weight and polarity, starch cannot be processed by the usual conversion technologies of plastics [42]. Additionally, heat and water sensitivity further complicate the use of starch for the modification of PLA. Accordingly, it is usually plasticized with water, glycerol, or other polar compounds capable of forming hydrogen bonds with starch [43,44,45]. However, thermoplastic starch (TPS) has rather poor mechanical properties like small stiffness, strength, and deformability, but the disadvantages like heat and water sensitivity, and larger viscosity, remain practically the same. On the other hand, native starch is a stiff material which can form strong hydrogen bonds with PLA thus reinforcing it in a similar way to inorganic fillers like calcium carbonate, talc, or kaolin. Only a few papers have been published on PLA/starch composites, but most of them deal with the treatment or the modification of starch in order to improve its adhesion to PLA [46,47,48]. In spite of their goal, unfortunately these works do not provide a detailed analysis of the interactions between the components and on local processes taking place during deformation. According to the best of our knowledge, no one has yet pointed out the differences or similarities between natural fibers and native starch as additives for PLA.

In accordance with the considerations presented above, the goal of this work was to prepare PLA/native starch composites and compare their properties to those modified with a natural reinforcement, sugarcane bagasse fiber. All components are derived from natural resources thus they are biodegradable with advantageous environmental impact. The similarity of their chemical structure also justifies comparison. The majority of publications on PLA/natural fiber composites claim weak interactions between the components, thus an attempt was made to modify them by the addition of a functionalized, maleated PLA polymer. Since both the natural fibers and starch are expected to increase stiffness and strength, the attention is focused mainly on the mechanical properties of the composites, i.e., stiffness, strength, and impact resistance, and efforts were made to explore the mechanism of the deformation and failure of the composites. Some comments on the relevance of the results for practice are also mentioned in the final section of the paper.

## 2. Results

The following sections summarize the results of the work and include some discussion of the results as well. The chemical and physical structures of the two modifying components are compared in the first section and then their effect on composite properties is shown in the next. The quantitative analysis of the extent of reinforcement is presented in the subsequent section, followed by the discussion of local deformation processes, as well as the consequence of the results for the possible practical application of the composites.

### 2.1. Structure of the Reinforcements

**Scheme 1:**
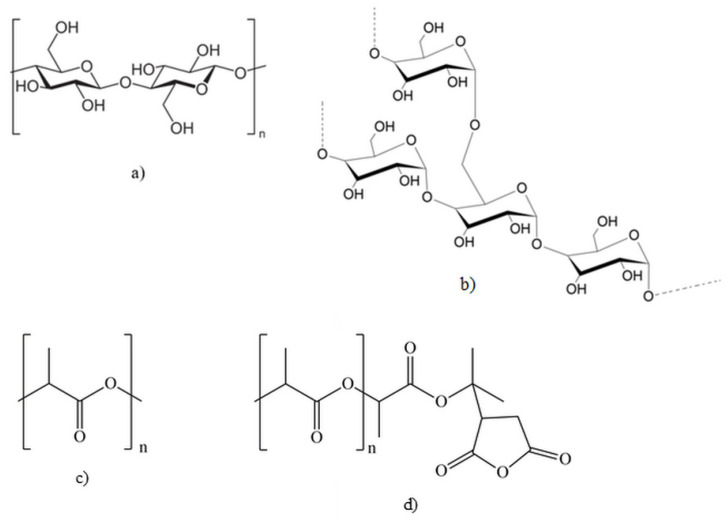
Chemical structure of the materials used in the study: (**a**) cellulose; (**b**) amylopectin (the structure of amylose is the same but without branching); (**c**) PLA; (**d**) MAPLA.

The chemical and physical structure of the reinforcement used strongly influence its effect on the matrix polymer and on the properties of the resulting composite [49]. The chemical structure of the two reinforcements used in this study shows strong similarities, but some differences as well. Both polymers are polysaccharides consisting of glucose units. The coupling of these units is different, cellulose contains β-glucosides [50], while starch consists of α units [41]. Cellulose molecules are linear, while starch chains are helical. The chain structure of the two polymers is shown in Scheme 1. Starch consists of linear amylose and branched amylopectin chains (only this latter is shown in the scheme). In spite of the differences mentioned, both polymers contain a large number of hydroxyl groups capable of forming relatively strong hydrogen bonds with PLA.

The phase structure of the two reinforcements also shows some similarities, both are semicrystalline materials containing an ordered, crystalline phase. Both the strong self-interactions and crystallinity result in large stiffness. However, while starch is a relatively pure material, cellulose fibers contain other components as well, mainly hemicellulose, lignin, and waxes [50,51]. The oriented chains located in cellulose crystals result in increased stiffness and strength in the direction of the orientation and smaller strength perpendicularly to it [52]. Such orientation does not exist in starch particles.

**Figure 1:**
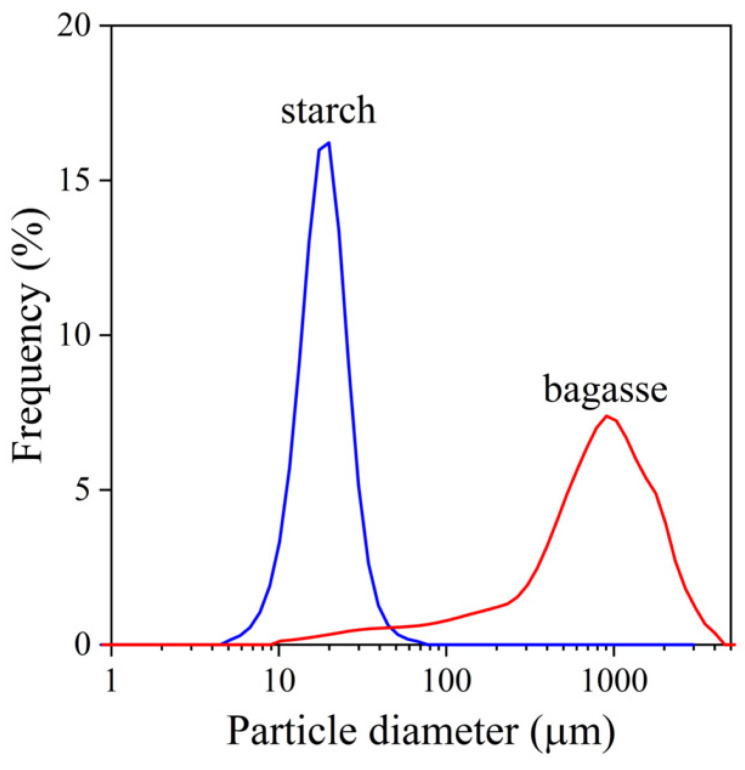
Particle size distribution of the additives as determined by laser light scattering.

Particle morphology is crucial for such materials used as reinforcements in polymer composites. Size, shape, and the extent of anisotropy, i.e., aspect ratio, are extremely important in the determination of deformation processes and final properties [53,54]. The size distribution of the two materials determined by laser light scattering is compared in Figure 1. The average size of starch particles is around 20 μm, while bagasse fibers are much larger, the most frequent size being around 900–1000 μm. However, since sugarcane bagasse fibers have an anisotropic shape, their size was also determined by microscopy. According to these measurements, the average length of the fibers is 2850 μm, their diameter 720 μm and thus their aspect ratio is around 5.1. Moreover, we must call attention to the fact here that the fibers change their size during processing, considerable attrition takes place and thus the final length of the fibers in PLA composites processed by extrusion and injection molding was 730 μm, their diameter 170 μm, and aspect ratio decreased to 3.0 [26]. Although considerable similarities can be found in the chemical structure of the two reinforcements, their physical structure, composition, and particle morphology differ considerably; it remains to be seen which factor determines the extent of reinforcement and composite properties.

### 2.2. Composite Properties

**Figure 2:**
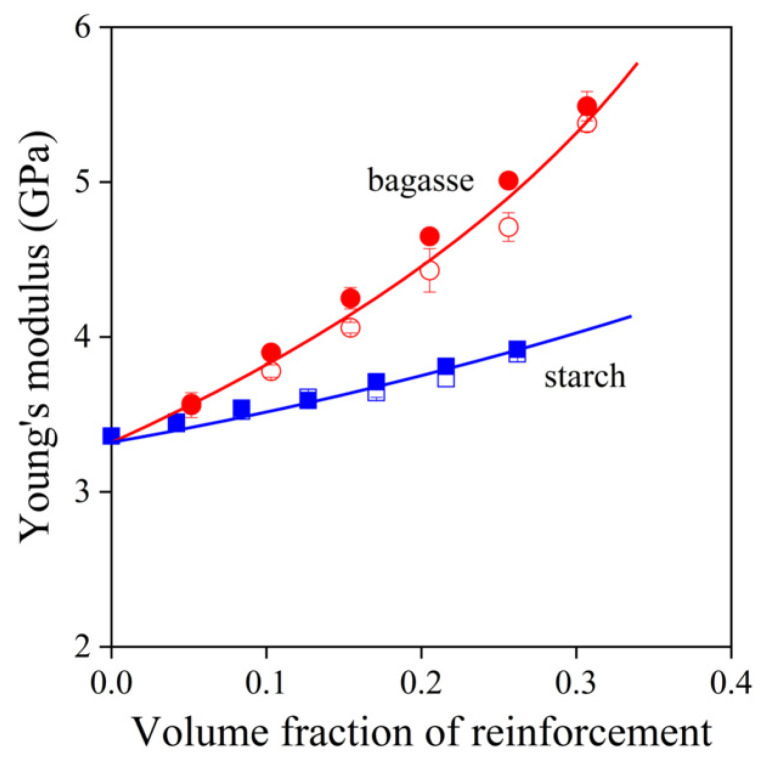
Composition dependence of the stiffness of PLA composites reinforced with starch or bagasse fibers. Effect of coupling. Symbols: (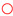, 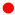) sugarcane bagasse fibers, (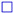, 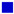) starch; empty symbols without MAPLA, full symbols with MAPLA.

Since fiber-reinforced materials are mostly used in structural applications, the attention is focused mainly on their mechanical properties. The stiffness of the composites containing various amounts of the two reinforcements is shown in Figure 2. Both reinforcements increase stiffness, but to considerably different extents, bagasse fibers have a much stronger reinforcing effect than starch. The stiffness of natural fibers was shown to cover a wide range from several 10 to several 100 GPa [50,55]; the direct measurement of the stiffness of the fibers used in this study resulted in the value of 25 GPa [51] that is in line with their reinforcing effect. Unfortunately, the direct determination of the stiffness of starch particles is very difficult, if not impossible, and we found only a single paper reporting the modulus value for starch, which was derived indirectly from the study of epoxy resin/starch and polycaprolactone/starch blends [56]. The value given was 2.7 GPa that cannot be correct since the addition of starch clearly increases the stiffness of neat PLA having a modulus of 3.3 GPa. 

We may assume that the inherent stiffness of starch is smaller than that of the sugarcane bagasse fibers, but the anisotropic particle geometry and the orientation of the fibers must also contribute to the larger stiffness of their composites. Using MAPLA as coupling agent in the composites does not improve or deteriorate stiffness that is not very surprising, since stiffness is not very sensitive to changes in interfacial adhesion [25,57].

**Figure 3:**
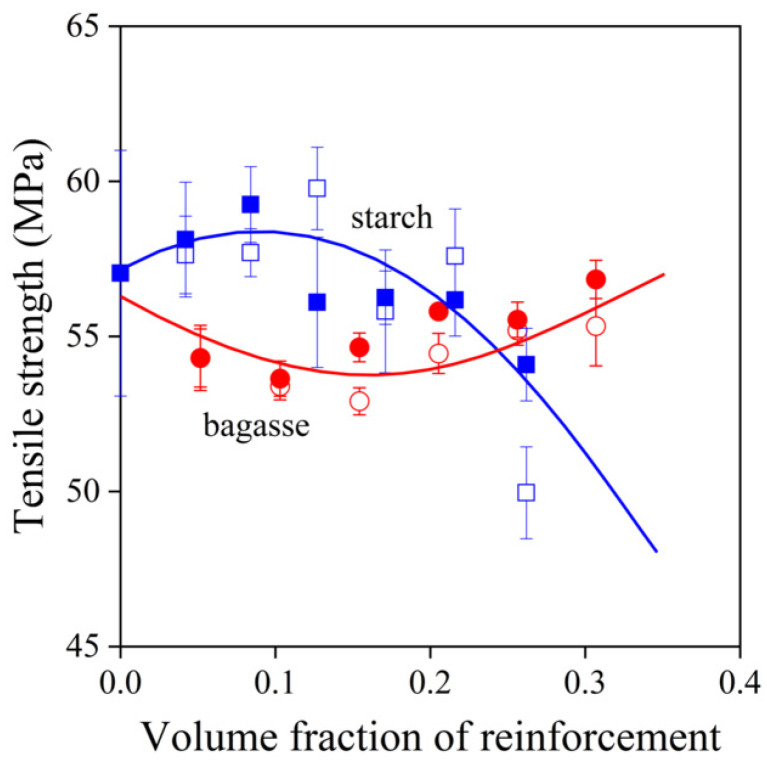
Effect of composition and coupling on the tensile strength of PLA composites. Symbols: (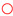, 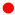) sugarcane bagasse fibers, (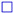, 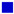) starch; empty symbols without MAPLA, full symbols with MAPLA.

The deformability of the composites (not shown) decreases marginally with an increasing amount of the additives independently of their type or coupling. The strength of the composites containing the two reinforcements is presented in Figure 3 as a function of additive content. The results are quite surprising, starch having a stronger reinforcing effect than the bagasse fibers, at least at smaller additive loadings. The orientation of the anisotropic fibers should lead to stronger composites similarly to stiffness. Obviously, other factors, most probably local deformation processes, play a significant role in the determination of composite strength. The much larger size of the fibers, even after attrition, facilitates debonding and leads to smaller strength. Although starch particles of around 20 μm size may also debond, but at a larger stress than the fibers having a size of one order of magnitude larger.

Another surprising result is that the MAPLA coupling agent practically does not affect the tensile strength of the composites either, although properties measured at larger deformations depend on interfacial adhesion quite strongly [57]. In spite of numerous claims published in the literature that the interaction between natural fibers and PLA is weak [28,29,31], we have proved earlier that relatively strong interactions develop in such composites [26,58]. Considering the similarities in the chemical structure of starch and bagasse fibers (large number of OH groups), the lack of any coupling effect is not surprising in the case of starch either. However, the relative influence of particle size and interfacial interactions must be considered in the further evaluation of the results.

**Figure 4:**
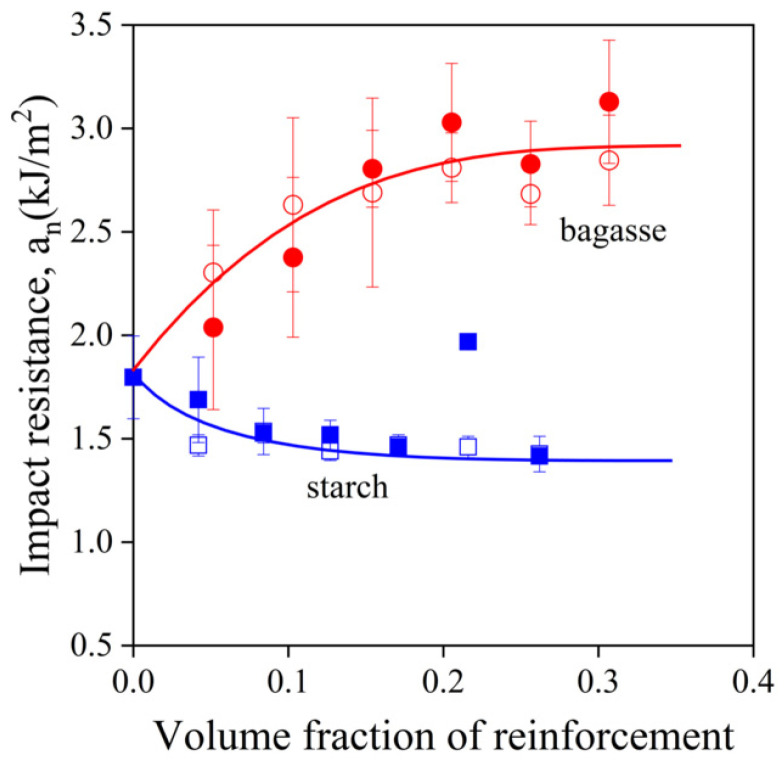
Dependence of the notched Charpy impact resistance of PLA composites containing natural reinforcements. Symbols: (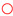, 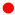) sugarcane bagasse fibers, (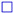, 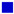) starch; empty symbols without MAPLA, full symbols with MAPLA.

Impact resistance is another mechanical property that is important for most structural materials; generally large strength and impact resistance is required from such materials used in practice [59,60]. The impact strength of the composites prepared in this study is presented in Figure 4. The effect of the two reinforcements differs considerably from each other. The addition of bagasse fibers doubles the impact strength of PLA, while the addition of starch decreases it slightly. The opposite effects must be caused by the different failure mechanism of the composites containing the two kinds of additives. The effect of coupling is very slight again. This small effect must be related to the interaction of the components and to the local deformation processes taking place during failure. The main factors determining composite properties are particle characteristics and to some extent interfacial adhesion. 

### 2.3. Reinforcement

The reinforcing effect of additives is difficult to assess based on the primary results of stiffness and especially strength values even though it is almost invariably done so. Reinforcement depends on several factors including the spatial arrangements of the fibers, their size, shape, and orientation as well as on interfacial interactions. Mathematical models give a quantitative estimation of the reinforcing effect of the most various second components. Such a model was developed earlier, which describes the composition dependence of tensile strength [61]. The model can be expressed as (Equation (1))

(1) $$\sigma_{T}=\sigma_{T0}\;\lambda^{n}\;\frac{1-\varphi_{f}}{1+2.5\;\varphi_{f}}\;\exp\left(B\;\varphi_{f}\right)$$

where *σ_T_* shows the true tensile strength of the composite; *σ_T_*_0_ is the same for the matrix, *φ_f_* shows the volume fraction of the second component, and *B* is the load-bearing capacity of the reinforcement. The latter depends on interfacial adhesion. True tensile strength (*σ_T_* = *σλ*, where *λ* = *L/L*_0_, is the relative elongation) expresses the change in the cross-section of the specimen during deformation and *λ^n^* takes into account strain hardening (*n* can be determined from matrix properties and characterizes the strain hardening tendency. Reduced tensile strength can be expressed by the rearrangement of Equation (1)

(2) $$\sigma_{Tred}=\frac{\sigma_{T}}{\lambda^{n}}\;\frac{1+2.5\;\varphi_{f}}{1-\varphi_{f}}=\sigma_{T0}\;\exp\left(B\;\varphi_{f}\right)$$

**Figure 5:**
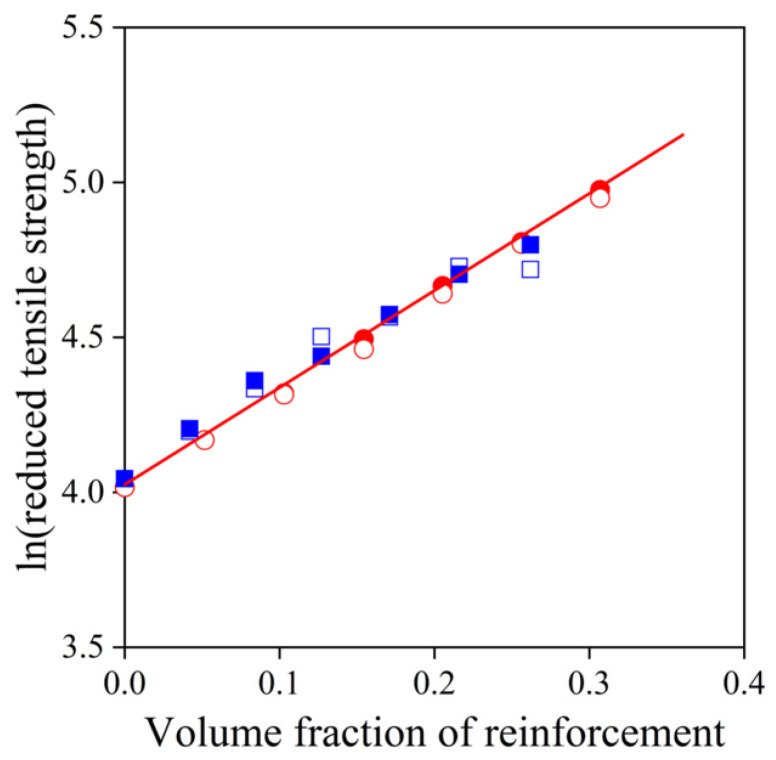
Reinforcing effect of the natural additives used in PLA composites. Tensile strength plotted in the linear representation of Equation (2). Effect of coupling. Symbols: (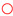, 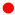) sugarcane bagasse fibers, (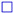, 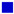) starch; empty symbols without MAPLA, full symbols with MAPLA.

The natural logarithm of tensile strength can be plotted against composition and the plot should result a straight line. The slope of the line expresses the reinforcing effect of the additives quantitatively. The tensile strength of the composites is plotted in the way indicated by Equation (2) in Figure 5. We obtain straight lines as expected verifying the validity of the approach and showing the lack of considerable structural effects.

The analysis indicates that the load-bearing capacity of the two reinforcements is similar in spite of the different primary values shown in Figure 3. The parameters determined by the fitting of the model to the experimental values are compiled in Table 1. The comparison of the data listed in the table shows the very slight overall effect of coupling as well. The similar reinforcing effect of the two additives might be surprising first but can be reasonably explained by the smaller size of starch particles increasing the value of parameter *B* and the orientation of bagasse fibers, which compensates for the negative effect of large size. 

The strength of interfacial adhesion is difficult to predict from the model because of the different size and shape of the particles, but it can be estimated by the reversible work of adhesion. This latter can be calculated from the surface tension of the components. The matrix of the composites was the same, and the surface tension of the two reinforcements is quite similar, 35 mJ/m^2^ was measured for starch, while 38 mJ/m^2^ for bagasse fibers by inverse gas chromatography leading to similar work of adhesions (99.4 mJ/m^2^ and 100.2 mJ/m^2^ respectively), i.e., strength of interfacial adhesion.

### 2.4. Deformation and Failure

The results presented above indicate that considerable differences exist in the properties of the composites prepared with the two kinds of reinforcements. Stiffness and impact resistance was larger for the PLA/bagasse fiber composites that is a slight contradiction in itself since larger stiffness is usually accompanied by smaller impact strength [62], while the strength of the PLA/starch composites exceeded that of the other set of materials. Since the strength of interfacial adhesion is similar, the main reason for the differences must lay in the particle characteristics of the additives and the local deformation processes initiated by them.

**Figure 6:**
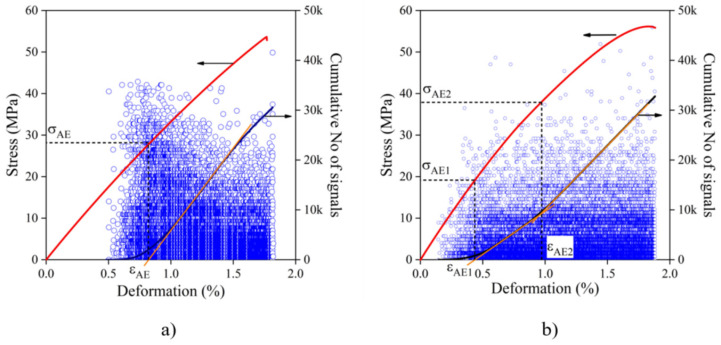
Results of the acoustic emission testing of PLA composites containing starch or bagasse fibers. Additive content: 20 vol%. Symbols: (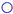) individual acoustic events, full lines: stress vs. deformation (left axis, red) and cumulative number of signal vs. deformation (right axis, navy) correlations: (**a**) starch, (**b**) sugarcane bagasse fiber.

Composites are heterogeneous materials containing components with dissimilar elastic properties. External load results in the development of local stress maxima, which initiate local deformation processes. These processes like the separation of the interface between the matrix and the reinforcement (debonding), and the fracture of the particles can be followed by various techniques including volume strain measurements, or by acoustic emission tests. The results of the latter measurements are presented in Figure 6 for two composites, one containing starch (Figure 6a) and the other prepared with bagasse fibers (Figure 6b). The small circles in the figure indicate individual acoustic events, while the two continuous lines show the stress vs. strain curve for reference (left axis), as well as the cumulative number of signal vs. deformation trace (right axis). This latter shows the total number of signals detected up to a certain deformation and its shape offers information about the process itself. Debonding is often accompanied by a saturation-like correlation [59], but the shape in itself does not allow the unambiguous identification of the dominating local deformation process [63].

The comparison of the two plots presented in Figure 6a,b reveals a number of similarities, but also differences. The amplitude, i.e., strength, of the signals indicated by the height of the points on the vertical axis is approximately the same, although signals with higher energy seem to be located in larger numbers at the first part of the measurement in the starch composite (Figure 6a). Both plots show that signals start to appear after a certain, critical deformation, which is larger for the starch composite than for the one containing bagasse. If debonding is the main local deformation process, this difference can be clearly explained by the smaller size of the starch particles, since debonding stress is inversely proportional to particle size [64]. The signals in Figure 6a seem to form only one group but their high amplitude and the shape of the cumulative number of signals trace indicate the occurrence of a second process besides debonding which may be the fracture of starch particles, although one would not expect this to happen and it needs further proof.

**Figure 7:**
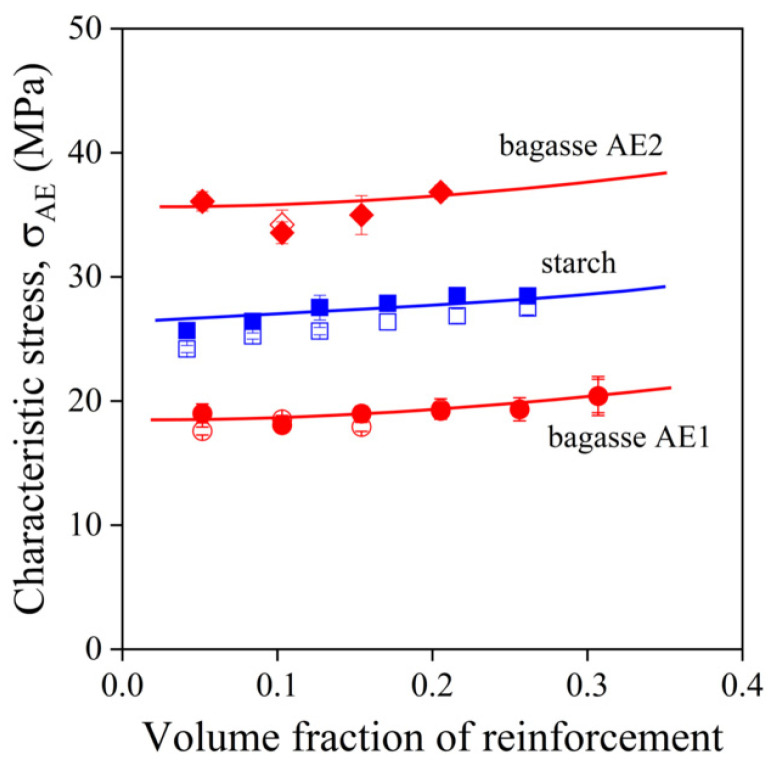
Composition dependence of characteristic stresses derived from acoustic emission testing. Symbols: (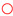, 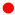) sugarcane bagasse fibers, σ_AE1_ (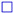, 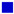) starch, σ_AE_, (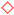, 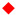) bagasse, σ_AE2_; empty symbols without MAPLA, full symbols with MAPLA.

The plotting of the cumulative number of signal traces allows the determination of characteristic stresses which can be assigned to the local processes taking place during the deformation of the specimen. The determination of these values from the plots is shown in Figure 6. Characteristic stresses determined for the processes detected, σ_AE1_ and σ_AE2_, are plotted against composition in Figure 7. Only one characteristic stress could be determined for the starch composites with any reliability. The first process in bagasse fiber composites is initiated at smaller stress and is unambiguously assigned to debonding. The larger stress obtained for starch is consistent with the explanation given above, i.e., debonding needs larger stress for smaller particles. In the case of the fiber-reinforced polymer the second process is mostly related to the fracture of the fibers [52,57,59]. 

Fracture is difficult to imagine for starch particles, but it is not impossible. The stress belonging to fiber fracture is much larger and can be clearly separated from the first process for the bagasse composites. The existence of a second, competitive process cannot be identified definitely for starch-filled composites but SEM micrographs may provide further evidence about the processes occurring during failure. 

**Figure 8:**
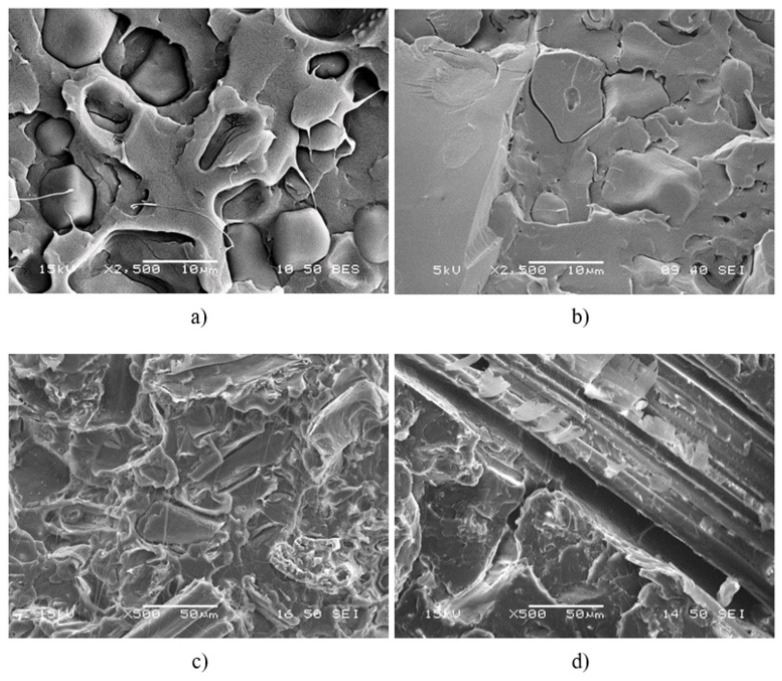
SEM micrographs recorded on the fractured surfaces of specimens broken during tensile testing: (**a**) starch, 20 wt%; (**b**) starch, 20 wt%, MAPLA; (**c**) sugarcane bagasse fiber, 20 wt%; and (**d**) bagasse fiber, 20 wt%, MAPLA.

SEM micrographs recorded on the fracture surface of specimens broken during tensile tests are presented in Figure 8 for samples containing starch or bagasse fibers with or without coupling. Considerable debonding can be observed in Figure 8a in the composite containing starch without coupling, while less debonding and the fracture of some particles are observed in Figure 8b in the presence of the coupling agent (MAPLA). One could draw the conclusion from these results that coupling is very efficient that would contradict all earlier conclusions. However, we must call the attention here to the fact that SEM micrographs cover only a very small area of the fractured surface of the specimen. Some micrographs prepared showed less debonding than in Figure 8a and even some broken particles in the absence of the coupling agent, and, on the other hand, larger number of debonded particles in its presence. Nevertheless, the micrographs verify the occurrence of two processes in these composites and identify them as debonding and particle fracture. Very similar micrographs were also recorded on composites containing the bagasse fibers, as shown by Figure 8c,d; the two processes, debonding and fiber fracture, can be clearly identified also in these micrographs. 

## 3. Discussion

The results presented in the previous sections clearly show that the main factors determining composite properties are particle characteristics and the inherent properties of the additives. Bagasse fibers increase stiffness to a larger extent than starch because of the larger stiffness of the fibers and their anisotropic particle characteristics. Strength is strongly influenced by local processes taking place during deformation. The smaller particle size of starch results in larger debonding stress (see also Figure 7, σ_AE1_) and thus larger composite strength compared to composites containing bagasse fibers. The presumably larger inherent strength of the fibers leads to considerably larger impact resistance for the PLA/bagasse fiber composites. Because of the similar interfacial interactions of the two reinforcements, interfacial adhesion is also very similar for the two (see Figure 5 and Table 1) and coupling has a very slight influence on properties. The limited efficiency of coupling can be explained also by the small number of functional groups and smaller molecular weight of the modified polymer as well as the small number of entanglements in PLA. The results are consistent and help to identify the main factors determining composite properties, but also the advantages and drawbacks of the two reinforcements.

**Figure 9:**
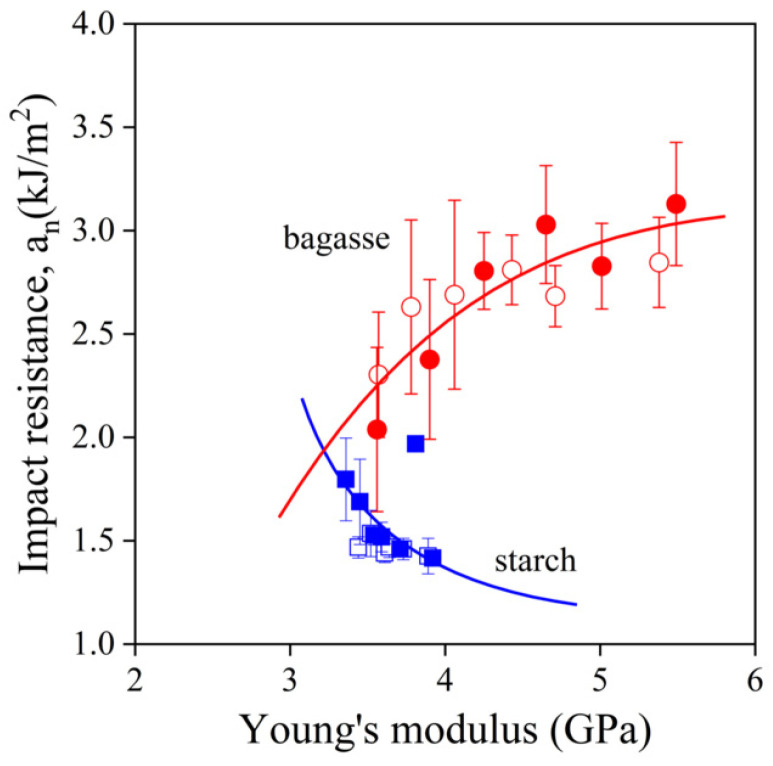
Correlation of the stiffness and impact strength of PLA composites reinforced with starch and sugarcane bagasse fibers. Opposing effects as a result of different local processes. Symbols: (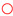, 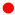) sugarcane bagasse fibers, (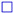, 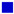) starch; empty symbols without MAPLA, full symbols with MAPLA.

One of the main requirements towards composites used as structural materials is large stiffness and impact resistance. The requirement is quite difficult to satisfy because they are inversely proportional in most structural materials, i.e., larger stiffness is usually accompanied by smaller impact strength. The two quantities are plotted against each other in Figure 9. The general tendency is indeed valid for the PLA/starch composites, but shows the opposite direction for the polymer reinforced with the bagasse fibers. The reason for the discrepancy is the relatively large inherent strength of the bagasse fibers and the fact that considerable fiber fracture takes place during the deformation and failure of its composites; in fact, fiber fracture might be the dominating local deformation process over debonding and it consumes considerable energy during failure. We must call attention here, though, that the absolute value of fracture resistance is not very large in any of the composites, and that much larger fracture strengths are required in certain applications. Nevertheless, bagasse fibers offer a reasonable overall performance over starch.

## 4. Materials and Methods

### 4.1. Materials

The matrix, poly(lactic acid) (Ingeo 4032D, M_n_ = 88,500 g/mol and M_w_/M_n_ = 1.8) was provided by NatureWorks LLC (Minnetonka, MN, USA). The extrusion grade polymer (<2% D isomer) has a density of 1.24 g/cm^3^, and its MFR is 3.9 g/10 min (190 °C and 2.16 kg). The production method of the maleic anhydride grafted PLA (MAPLA) coupling agent was described earlier in detail [25]. The Ingeo 3251D grade PLA (NatureWorks LLC, Minnetonka, MN, USA; MFR: 35 g/10 min at 190 °C and 2.16 kg load) was used for the grafting reaction. The reactive extrusion was carried out in a Brabender LabStation (Brabender GmbH, Duisburg, Germany) single screw extruder with the temperature profile of 175–180–185–190 °C. The screw speed was set to 12 rpm. 2 wt% maleic anhydride and 2 wt% Luperox 101 peroxide were added to the reaction mixture. MAPLA was characterized by NMR (Varian NMR System, Agilent Technologies, Inc., Santa Clara, CA, USA), however, it was not purified; it was applied as produced in the reactive extrusion process.

The bagasse fibers used as reinforcement were obtained from a sugar mill in Sidoarjo, Indonesia. The chemical composition of the fibers was determined by the van Soest method. The detailed description of the method can be found in the paper of van Soest [65]. According to the method, hemicellulose content is determined after treatment with an acidic detergent solution, the amount of cellulose by treating the fibers with sulfuric acid of 72 wt% concentration and lignin by burning the sample in an oven. The fibers contained 47% cellulose, 35% hemicellulose, 15% lignin, and 3% ash. The fibers were dried and sieved before extrusion. Fiber characteristics were determined by digital optical microscopy (Keyence VHX 5000, Keyence Co., Osaka, Japan). The native starch was derived from corn and was purchased from Hungrana Kft., Hungary. The amount of the additives in the polymer changed from 0 to 30 wt% in 5 wt% steps. The coupling agent (MAPLA) was added to the matrix polymer in 0.1:1 ratio calculated for the weight of starch or sugarcane bagasse fibers.

### 4.2. Sample Preparation

The modifying components were dried in a Memmert UF450 type oven (Memmert GmbH, Schwabach, Germany) before extrusion (bagasse fibers for 4 h, and starch for 24 h at 105 °C) to eliminate their residual moisture content. PLA and MAPLA were dried together in a vacuum oven at 100 °C and 150 mbar pressure. A Brabender DSK 42/7 (Brabender GmbH, Duisburg, Germany) twin-screw compounder was employed for the appropriate homogenization of the components at the set temperatures of 170–180–185–190 °C and the rate of homogenization of 40 rpm. The granules were injection molded into tensile bars according to the ISO 527 1A standard using a Demag IntElect 50/330-100 electric injection molding machine. The temperature profile of the barrel was the same as in extrusion, while injection pressure (800–1200 bar) depended on the amount of the reinforcement added. The value of the most important processing parameters were: injection speed: 50 mm/s; holding pressure: 650–800 bar; holding time: 15 s; cooling time: 45 s. The temperature of the mold was set to 20 °C. The specimens were kept under ambient conditions (23 °C, 50% RH) until further testing.

### 4.3. Characterization

An Instron 5566 universal testing machine (Instron, Norwood, MA, USA) was used for the characterization of tensile properties with a gauge length of 115 mm and 5 mm/min crosshead speed. An acoustic emission (AE) equipment was used simultaneously with tensile testing to detect local deformation processes. The specific equipment used was a Sensophone AED 404 apparatus manufactured by Geréb és Társa Ltd. (Budapest, Hungary). A single piezoelectric sensor (150 kHz resonance frequency) was attached to the center of the specimen. The threshold level of detection was set to 25 dB. A Ceast Resil 5.5 impact tester (Ceast spa, Pianezza, Italy) was used for the determination of Charpy impact strength (ISO 179 standard at 23 °C with 2 mm notch depth) of the specimens. Fracture surfaces were studied by scanning electron microscopy (Jeol JSM 6380 LA, Jeol Ltd., Tokyo, Japan). Micrographs were recorded on surfaces created during tensile and fracture testing, respectively. Accelerating voltage (5 and 15 kV) and distance to the sample changed according to magnification and the quality of the image. Detectors were used both for secondary and back-scattered electrons. Surfaces were sputtered with gold before the recording of micrographs from the composites. The particle size and size distribution of the modifying components were determined by laser light scattering using a Horiba LA 950 A2 (Horiba, Kyoto, Japan) analyzer. The size, size distribution, and aspect ratio of the bagasse fibers were also determined after processing by digital optical microscopy (Keyence VHX 5000, Keyence Co., Osaka, Japan). The polymer was dissolved in tetrahydrofuran (Molar Chemicals Kft., Hungary) to extract the fibers from the composites.

## 5. Conclusions

The comparison of the properties of PLA composites reinforced with native starch and sugarcane bagasse fibers shows that the two additives influence properties differently. The analysis of their chemical and physical structure indicates that the former is quite similar, while considerable differences exist in the latter. Particle characteristics differ even more and are shown to influence properties considerably. The strength of interfacial adhesion, and thus the extent of reinforcement, are similar because of the similarities in the chemical structure of the reinforcements. Relatively strong interfacial adhesion develops between the components which renders coupling inefficient. Dissimilar particle characteristics, on the other hand, influence local deformation processes considerably. The smaller particle size of starch results in larger debonding stress and thus larger composite strength. Besides debonding, considerable fiber or particle fracture also takes place during the failure of the composites. The larger inherent strength of the bagasse fibers leads to larger energy consumption during fracture and increased impact resistance. Although the environmental benefit of the prepared biocomposites is very similar, the overall performance of the bagasse fibers reinforced PLA composites is better than that offered by the PLA/starch composites.

## Figures and Tables

**Table 1:** Parameter (*B*) characterizing the load-bearing capacity of the reinforcements studied (see Equations (1) and (2)).

Reinforcement	Coupling	*σ_T_*_0_ (MPa)	Parameter *B*	R^2 a^
Starch	–	58.2	3.11 ± 0.16	0.9896
	+	59.2	2.84 ± 0.12	0.9910
Bagasse	–	55.0	3.07 ± 0.04	0.9992
	+	55.1	3.14 ± 0.03	0.9994

^a^ determination coefficient indicating the goodness of the fit.

## Data Availability

The raw/processed data required to reproduce these findings cannot be shared at this time due to legal or ethical reasons.
